# Risankizumab and guselkumab for psoriasis: a 1-year real-world practice indirect comparison^☆^

**DOI:** 10.1016/j.abd.2024.05.005

**Published:** 2025-01-09

**Authors:** Leyla Baykal Selçuk, Hande Ermiş Akkuş, Burak Akşan, Deniz Aksu Arıca

**Affiliations:** a Department of Dermatology and Venereology, Karadeniz Technical University Faculty of Medicine, Trabzon, Turkey; b Ankara Sincan Training and Research Hospital, Ankara, Turkey; c Department of Dermatology and Venereology, Giresun University Faculty of Medicine, Giresun, Turkey

**Keywords:** Biological products, Guselkumab, Psoriasis, Risankizumab

## Abstract

**Background:**

Psoriasis is a chronic inflammatory skin disease with a genetic predisposition and autoimmune component, often treated with immunomodulators such as biologic therapies.

**Objectives:**

In this study, the authors evaluated the effectiveness and safety of two of these over a 52-week treatment period.

**Methods:**

A double-center retrospective cohort study was conducted, enrolling patients with moderate to severe psoriasis who received either guselkumab or risankizumab at dermatology clinics for a minimum of 52-weeks.

**Result:**

Out of the 90 patients enrolled in the study, 49 (54.4%) received guselkumab, while 41 (45.6%) received risankizumab. Regarding therapy efficiency, there was no statistically significant difference in PASI90 and PASI100 at week 4 between the two groups (p = 0.428, p = 0.750, respectively). By week 16, PASI90 responses were higher in the guselkumab group (p = 0.039). However, there was no difference in PASI100 response at week 16 (p = 0.957). At weeks 24 and 52, PASI90 and PASI100 responses were similar in both groups. Our results demonstrated that both guselkumab and risankizumab were effective in patients who had previously failed other biologics. Clinical outcomes in both the guselkumab and risankizumab groups had remained unaffected during prior biologic treatments, including anti-TNF, anti-IL17, and/or anti-IL12/23. Treatments yielded consistent outcomes regardless of factors such as obesity, gender, and comorbidities.

**Study limitations:**

The small sample size.

**Conclusions:**

Our results demonstrated that both guselkumab and risankizumab were effective in patients who had previously failed other biologics. Clinical outcomes in both the guselkumab and risankizumab groups had remained unaffected during prior biologic treatments, including anti-TNF, anti-IL17, and/or anti-IL12/23. Treatments yielded consistent outcomes regardless of factors such as obesity, gender, and comorbidities.

## Introduction

Psoriasis is a chronic inflammatory skin disease with a genetic predisposition and autoimmune component, usually treated with immunomodulators such as biologic therapies. The disease spectrum mainly affects the skin and joints, correlating with various comorbidities, particularly cardiometabolic conditions.^1^, ^2^ Substantial progress in the field of biologic treatments has led to the development of a new category of human monoclonal antibodies, guselkumab and risankizumab, targeting the p19 subunit of IL23, which received approval from the US Food and Drug Administration (FDA) in 2017 and 2019, respectively.^2^ Their efficacy and safety have been demonstrated in various clinical trials, including VOYAGE-1 and VOYAGE-2^3^, ^4^ and ECLIPSE^5^ for guselkumab, as well as UltIMMa-1 and UltIMMa-2,^6^ IMMERGE^7^ and IMMVENT^8^ for risankizumab.

Real-life studies are crucial for verifying the efficacy and safety of recently approved biologics for psoriasis in a more diverse patient population, often excluded from clinical trials. Insights into the practical use of these two agents in psoriasis are being shared through the reporting of real-life data from various countries. Despite this, real-world data comparing guselkumab and risankizumab effectiveness and safety are quite limited. In the literature, Ruggiero et al. and Viopulus et al. compared the efficacy and safety of these biologics and established that both showed similar efficacy for 44 and 24 weeks respectively.^9^, ^10^ In our study, the authors evaluated the effectiveness and safety of both drugs over a 52-week treatment period.

## Materials and methods

A double-center retrospective cohort study was conducted, enrolling patients with moderate to severe psoriasis who received either guselkumab or risankizumab at dermatology clinics from August 2022 to March 2023. Inclusion criteria comprised patients over 18 years of age, diagnosed with moderate-to-severe psoriasis and treated with either guselkumab or risankizumab for a minimum of 52 weeks. Patients treated with guselkumab received 100 mg doses at weeks 0, 4, and then every 8 weeks thereafter, while those treated with risankizumab received 150 mg doses at weeks 0, 4, and then every 12 weeks thereafter. Psoriasis severity was assessed at weeks 4, 16, 28, and 52 through Physicians' Global Assessment (PGA). PASI90 (Psoriasis Area and Severity Index) and PASI100 scores were also conducted.

Demographic data, comorbidities (hypertension, diabetes mellitus, dyslipidemia, obesity, non-alcoholic fatty liver disease), psoriasis subtype, affected body areas, presence of Psoriatic Arthritis (PsA), history of previous systemic therapies and biologic therapies, treatment durations, treatment efficacy, adverse reactions, as well as baseline and follow-up blood test results (complete blood count, transaminases, creatinine, azotemia, glycemia, erythrocyte sedimentation rate, C-reactive protein, total cholesterol, and triglyceride levels) were collected retrospectively.

The study was conducted in accordance with the Declaration of Helsinki after receiving approval from the local ethical committees of the two participating universities.

### Statistical analysis

Statistical analysis was carried out using IBM SPSS version 25 for Windows. Results were expressed as numbers (n) and percentages (%) for descriptive data. Chi-Square analysis was employed for comparing categorical variables. The normal distribution was assessed using the Kolmogorov-Smirnov test. Student *t*-test was applied to compare normally distributed constant variables, while the Mann-Whitney *U* test was used for non-normally distributed variables. Correlation analysis was performed using Spearman’s correlation test. The relationship between the parameters was analyzed by either Pearson or Spearman analysis. A p-value of < 0.05 was considered statistically significant.

## Results

The demographics and characteristics of the study population are summarized in Table 1. Out of the 90 patients enrolled in the study, 49 (54.4%) received guselkumab, while 41 (45.6%) received risankizumab. The guselkumab group comprised 29 females (59.1%) and 20 males, whereas the risankizumab group consisted of 17 females (41.4%) and 24 males (58.6%). There was no statistically significant difference between genders in terms of biologic type (p = 0.094). Particularly, among comorbidities hypertension (18.4% vs. 17.1%,) was the most common condition but no significant differences were observed between the two groups.

**Table 1:** Demographic and clinical data of study population.

Variables	Guselkumab (n = 49)	Risankizumab (n = 41)	p-value
Gender, n (%)			0.094
Female	29 (59.2)	17 (41.5)	
Male	20 (40.8)	24 (58.5)	
Mean age mean ± std. dev	43.02 ± 14.09	45.34 ± 15.31	0.242
Disease duration mean ± std. dev	15.49 ± 11.32	15.54 ± 9.84	0.149
Psoriatic arthritis, n (%)	16 (32.7)	8 (19.5)	0.160
Obesity, n (%)	16 (32.7)	14 (35.9)	0.915
Comorbidities, n (%)			
None	39 (79.6)	22 (54)	0.009
> 1	10 (20.4)	19 (46.3)	
Diabetes	5 (10.2)	7 (17.1)	0.340
Hypertension	9 (18.4)	7 (17.1)	0.873
Dyslipidemia	6 (12.2)	5 (12.2)	0.994
Metabolic syndrome	5 (10.2)	6 (14.6)	0.523
Fatty liver	2 (4.1)	5 (12.2)	0.152
Scalp involvement, n (%)	30 (61.2)	26 (63.4)	0.831
Genital involvement, n (%)	13 (26,5)	12 (29.3)	0.773
Bionaive, n (%)	25 (51.0)	9 (22.0)	0.005
Previous IL-17 failure, n (%)	10 (20.4)	15 (36.6)	0.088
Number of previous biologics, n (%)			
1	28 (40.8)	20 (48.8)	
2	11 (22.4)	11 (26.8)	
3	2 (4.1)	8 (19.5)	
4	8 (16.3)	2 (4.9)	
PASI mean ± SD			
Before treatment	18.24 ± 7.25	17.04 ± 6.27	
After treatment	0.87 ± 1.46	0.82 ± 0.97	
PGA mean ± SD			
Before treatment	3.47 ± 0.54	3.46 ± 0.55	0.940
After Treatment	0.60 ± 0.70	0.56 ± 0.55	0.958
Treatment Responses			
At week 4			
PASI90	28 (57.1)	20 (48.8)	0.428
PASI100	6 (12.2)	4 (9.8)	0.750
At week 16			
PASI90	47 (95.9)	33 (80.5)	*0.039*
PASI100	17 (34.7)	14 (34.1)	0.957
At week 24			
PASI90	48 (98.0)	40 (97.6)	1.000
PASI100	28 (57.1)	23 (56.1)	0.921
At week 52			
PASI90	48 (98.0)	38 (92.7)	0.327
PASI100	27 (55.1)	22 (53.7)	0.891

The rate of being bioexperienced was significantly higher in the guselkumab group (p = 0.005). No remarkable difference was observed in terms of previous IL-17 failure (p = 0.103). The ratios of scalp and genital involvement were similar in both groups (p = 0.831 and p = 0.773, respectively).

**Fig. 1:**
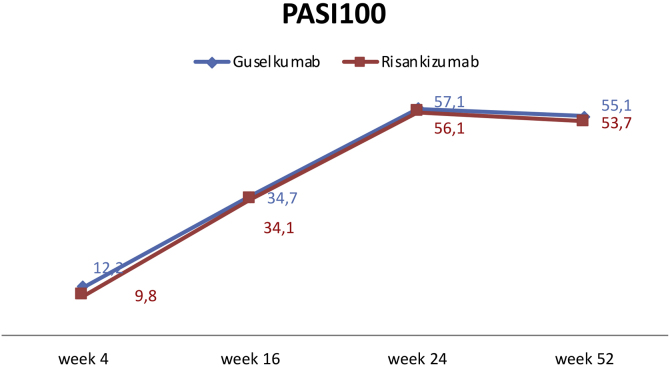
Rates of PASI100 responses of patients in groups of guselkumab and risankizumab.

Regarding therapy efficiency, there was no statistically significant difference in PASI90 and PASI100 at week 4 between the two groups (p = 0.428, p = 0.750, respectively). By week 16, PASI90 responses were higher in the guselkumab group (p = 0.039). However, there was no difference in PASI100 response at week 16 (p = 0.957). At weeks 24 and 52, PASI90 and PASI100 responses were similar in both groups (see Table 1 and Fig. 1).

When evaluating PASI100 responses in patients with scalp and genital area involvement, the rate of PASI100 response in the guselkumab arm was significantly higher in the 4th week among the patients with scalp involvement (p = 0.006). However, this difference was not sustained at weeks 16 and 24. No differences were found in the PASI100 responses at the 4^th^, 16^th^, and 24^th^ weeks among the patients with genital area involvement.

### PASI100 responses in different patient subgroups

No significant difference was shown in the PASI100 rates of guselkumab and risankizumab in bionaive patients compared to bioexperienced patients at weeks 4, 16, 24, and 52. Similarly, there was no significant difference in PASI100 rates at 4, 16, 24, and 52 weeks in obese patients, patients with comorbidities, and patients with psoriatic arthritis (as shown in Table 2).

**Table 2:** Comparison of PASI100 responses with risankizumab and guselkumab treatment according to patient's comorbid conditions, genders and previous treatments.

PASI100 responses	Guselkumab	Risankizumab	p-value
Gender: Female n (%)			
Week 4	3 (10.3)	2 (11.8)	0.881
Week 16	9 (31.0)	7 (41.2)	0.534
Week 24	15 (51.7)	10 (58.8)	0.762
Week 52	15 (51.7)	9 (52.9)	1.000
Gender: Male, n (%)			
Week 4	3 (15.0)	2 (8.3)	0.488
Week 16	8 (40.0)	7 (29.2)	0.532
Week 24	13 (65.0)	13 (54.2)	0.547
Week 52	12 (60.0)	13 (54.2)	0.766
Bionaive patients, n (%)			
Week 4	5 (20.0)	2 (22.2)	0.732
Week 16	11 (44.0)	5 (55.6)	1.000
Week 24	16 (64.0)	6 (66.7)	1.000
Week 52	15 (60.0)	5 (55.6)	1.000
Obese patients, n (%)			
Week 4	3 (18.8)	0 (0.0)	0.088
Week 16	5 (31.2)	3 (21.4)	0.689
Week 24	10 (62.5)	7 (50.0)	0.713
Week 52	10 (62.5)	7 (50.0)	0.713
Patients with > 1 Comorbidity, n (%)			
Week 4	1 (10)	3 (15.8)	0.297
Week 16	5 (50)	7 (36.8)	0.694
Week 24	6 (60)	12 (63.2)	1.000
Week 52	5 (50)	12 (63.2)	0.694
Psoriatic arthritis, n (%)			
Week 4	1 (6.2)	1 (12.5)	0.451
Week 16	7 (43.8)	2 (25.0)	0.794
Week 24	9 (56.2)	5 (62.5)	1.000
Week 52	9 (56.2)	5 (61.5)	1.000
Patients with anti IL17 treatment failure			
Week 4	0 (0.0)	2 (13.3)	0.355
Week 16	4 (40.0)	5 (33.3)	1.000
Week 24	4 (40.0)	6 (40.0)	0.798
Week 52	4 (40.0)	6 (40.0)	1.000
Biologic treatment failure > 2, n (%)			
Week 4	0 (0)	1 (10.0)	0.479
Week 16	3 (30.0)	3 (30.0)	1.000
Week 24	5 (50.0)	4 (40.0)	1.000
Week 52	5 (50.0)	4 (40.0)	1.000

Two patients treated with guselkumab (4.1%) discontinued treatment due to secondary unresponsiveness. In the risankizumab arm, two patients (4.9%) discontinued treatment due to secondary unresponsiveness, while one patient (2.4%) dropped out due to exacerbation of joint complaints.

When the authors looked at the rates of side effects occurring under medication, they were similar in both treatment arms (guselkumab 20.4%, risankizumab 24.4%) (Table 3). The most commonly observed side effects were, respectively, pharyngitis, flu-like illness, and headache. In both risankizumab and guselkumab groups, one patient experienced a flare of previously known psoriatic arthritis and received additional methotrexate for the control of psoriatic arthritis. Both of the patients were bioexperienced.

**Table 3:** Adverse reactions due to guselkumab and risankizumab.

	Guselkumab (n = 49)	Risankizumab (n = 41)
Any adverse event, n (%)	10 (20.4%)	10 (24.4%)
Serious adverse events, n (%)*	1 (2.1%)	‒
Severe adverse events, n (%)	‒	‒
Adverse events leading to drug discontinuation, n (%)	‒	‒
Injection side reaction, n (%)	1 (2.1%)	‒
Nasopharengitis, n (%)	4 (8.1%)	4 (9.8%)
Flu-like illness, n (%)	3 (6.1%)	3(7.3%)
Headache, n (%)	2 (4.1%)	2 (4.9%)
Serious infections, n (%)	‒	‒
Flare of psoriatic arthritis, n (%)	1 (2.1%)	1(2.4%)
Requirement of additional conventional therapy/with MTX) n (%)	1 (2.1%)	1(2.4%)
Major adverse cardiovascular event, n (%)	‒	‒
Malignancies, n (%)	‒	‒
Malignancies excluding non-melanoma skin cancer, n (%)	‒	‒
Serious hypersensitivity, n (%)	‒	‒
Deaths, n (%)	‒	‒

## Discussion

The efficacy and safety of guselkumab and risankizumab, two selective IL‐23 inhibitors, have been demonstrated in several phase-III trials. The effectiveness of guselkumab was reported as 73.3% in PASI90 and 37.4% in PASI100 responses in the 16th week of the phase 3 VOYAGE-1.^3^ In VOYAGE-2, guselkumab demonstrated effectiveness by attaining PASI90 in 70% of cases and achieving PASI100 in 34.1% of cases.^4^ In our study, these rates were higher for PASI90 at 95.9% and lower for PASI100 at 34.7%, respectively, at week 16.

In UltIMMa-1 and UltIMMa-2; the effectiveness of risankizumab was reported as 74.8% for PASI90 and 50.7% for PASI100 at the 16^th^ week of the UltIMMa-2 study.^6^ In our study, these rates were higher for PASI90 at 80.5% and lower for PASI100 at 34.1%. Addressing the real-life data, Fougerousse et al. conducted a retrospective multicentric study with 194 psoriatic patients who received guselkumab. At week 16, 50.6% of patients achieved PASI90, and 38.3% achieved PASI100.^11^ Although the PASI100 response ratio mirrored our study, the PASI90 response rate was lower compared to our research. Benhadou et al. showed in 112 psoriatic patients receiving guselkumab slightly lower PASI90 (55.4%) and similar PASI100 (32.1%) responses at week 16 compared to our study.^12^ Regarding outcomes over an extended period, Galuzzo et al. reported PASI 90 and 100 responses of 78.9% and 63.2%, respectively, at 12 months with guselkumab.^13^ Despite a superior PASI90 response at week 52 in our study, the PASI100 ratio was lower compared to the mentioned study.

Hansel et al. conducted a 16-week retrospective study, PASI-100 and PASI-90 were achieved by 49.1% and 63.2%, respectively using risankizumab.^14^ Mastorino et al. reported the effectiveness of risankizumab PASI90 and PASI100 as 53% and 32% at weeks 16, respectively, and 82% and 73% at week 52.^15^ Gkalpakiotis et al. followed 154 patients undergoing risankizumab, PASI90 and PASI100 responses of 82.4% and 67.6%, respectively, at week 52.^16^ Our results were comparable to this study with a ratio of 93% and 53.7% of PASI90 and PAS100 responses, respectively, at week 52. Garguilo et al. examined the clinical outcomes of 131 patients treated with risankizumab, revealing PASI90 and PASI100 response rates of 55.73% and 36.64% at week 16, and 78.63% and 61.10% at week 52.^17^ Hansel et al. showed the efficacy of risankizumab by week 52, 85.5% PASI90 and 60% PASI100.^18^

Additionally, risankizumab’s efficacy was evaluated in patients who initially failed guselkumab. Patients treated with risankizumab showed lower sPGA scores after both 4 and 12 months compared to their baseline sPGA scores. 1scores 4 months of risankizumab, 46% of patients showed of an sPGA score of 0/1, increasing to 90% at 12 months.^19^

Elgard et al. reported in patients receiving guselkumab PASI90 as 48% and PASI100 as 44% at the 24^th^ week, while in patients receiving risankizumab, PASI90 was reported as 55.6%, and PASI100 as 38.9%.^20^ Ruggiero et al. compared the efficacy and safety of guselkumab and risankizumab and established that both drugs showed similar efficacy (PASI90 and PASI100) without significant differences.^9^ In their study, PASI100 responses were received in 47.2% of those who received guselkumab and in 46.8% of patients using risankizumab at the 44^th^ week, and these results were lower when compared to the responses obtained in our study at the 52^nd^ week.

Gerdes et al. followed 303 patients who received guselkumab for 52 weeks.^21^ By week 52, 78.4% (n = 192), 62.9% (n = 154) and 40.4% (n = 99) of the study cohort achieved PASI75, PASI90 and PAS100 responses, respectively. Contrary to our study results, biologic-naïve patients and patients who did not receive IL-17 inhibitor prior to guselkumab displayed better improvement in PASI scores compared to bio-experienced and ani-IL-17-experienced patients, respectively.^21^

Benhadou et al. established that being bioexperienced and being bionaive did not affect treatment outcome measures with guselkumab at week 16.^12^ On the other hand, Hansel and colleagues demonstrated in their research that, under risankizumab therapy, individuals with previous biologic exposure achieved PASI100 more frequently at both week 36 and week 52 than -bionaïve patients.^18^ Conversely, Galuzzo et al., reported that the number of comorbidities and previous biologic failure have negative ramifications of PASI responses.^13^ In the study by Vaiopoulos et al., significantly higher rates of PASI100 response were found in biologic-naive patients undergoing risankizumab and guselkumab treatment compared to non-naive patients (89.5 vs. 76.9%).^10^ The researchers observed that obesity did not affect the PASI100 response, conversely, patients without dyslipidemia had better skin responses. There was no difference in the Disease Activity Index for Psoriatic Arthritis scores between the medication groups in patients with and without psoriatic arthritis. Also, they found scalp psoriasis showed a rapid improvement starting from week 4 while palmoplantar psoriasis, nail psoriasis and psoriatic arthritis showed even though significant but slower improvement starting from week 16.^10^ Gerdes et al. also reported the high efficacy of guselkumab in scalp, palmoplantar, and genital psoriasis.^21^ Generally said, there was no difference in treatment response with risankizumab among those with specific area involvement, in an Italian study.^15^ Garguilo et al.'s study substantiated the previous one, particularly regarding the efficacy of risankizumab among patients, irrespective of specific area involvement.^17^

According to the study of Hansel et al., higher PASI75, 90 and 100 responses were established among patients whose BMI was lower than 25.^14^ In Gerdes et al.’s study, while the percentage of patients reaching PASI ≤ 1 was lower in individuals with a higher BMI, there was an elevated proportion of PASI ≤ 1 responders observed across all BMI categories from week 28 to week 52.^21^ This may indicate the sustainable and increasing efficacy of guselkumab in individuals with obesity. Accordingly, Galuzzo et al. reported that the presence of obesity did not emerge as a predictor of PASI response.^13^ Likewise, a multicenter study conducted in the Czech Republic revealed that both BMI and prior biologic therapy had no impact on PASI90 and PASI100 responses during risankizumab treatment.^16^ Our study demonstrated that obesity, being biologic-naïve or prevalence of PsA did not alter the effectiveness of guselkumab and risankizumab treatments.

Similarly to our study, Ruggiero et al. reported two patients who discontinued guselkumab due to worsening of PsA, whose joint complaints were already unresponsive to anti-TNF and anti-IL17.^1^ In line with the literature, the authors did not observe any tuberculosis activation in our study group.

Shu et al. conducted a pharmacovigilance study for risankizumab and reported significant adverse effects including myocardial infarction, thrombosis, and arterial occlusive disease.^22^ However, these effects were of weak clinical priority and mostly occurred in the first 3 months of therapy according to analyses. Although there were debates about adverse effects due to risankizumab, in our study, and as well as in Ruggiero et al.'s study, no serious adverse effects were observed.^1^

Selective targeting of the IL-23 pathway was not found to increase the risk of opportunistic infections, tuberculosis activation, oral candidiasis, or inflammatory bowel disease.^23^ After 5 years of follow-up of the 1721 patients (>7100 patient-years) who were treated with guselkumab, 32 patients had malignancies excluding NMSC (0.45/100 patient-years). This rate was comparable to the malignancy rate excluding NMSC (0.68/100 patient-years) in the Psoriasis Longitudinal Assessment and Registry.^24^ In a real-world study from Italy, among 307 patients treated with guselkumab, adverse effects were reported in 10 patients (3%), including one patient with transient ischemic attack, and two patients discontinued the drug due to erythroderma and malaise.^25^ Gerdes et al. observed (n = 303) four (1%) treatment-related serious adverse effects, i.e., bronchitis, EBV infection, malignant neoplasm, and pemphigoid.^21^ In the Czech study, risankizumab was discontinued in one participant due to colorectal cancer and in another due to Morbus Morbihan, with a potential link to risankizumab that could not be ruled out.^16^

The relatively small sample size and the retrospective nature of the study and the limited follow-up period may limit the generalizability of our results.

## Conclusions

Our results showed that both guselkumab and risankizumab were effective in patients who previously failed other biologics. Clinical outcomes in both the guselkumab and risankizumab groups remained unaffected by prior biologic treatments, such as anti-TNF, anti-IL17, and/or anti-IL12/23. Treatments yielded consistent outcomes regardless of factors like obesity, gender, and comorbidities. Our study also showed both guselkumab and risankizumab as safe treatment options, with the most frequently reported adverse events being pharyngitis, flu-like illness, and headache in the treatment groups. None of these adverse events required treatment discontinuation.

## Financial support

None declared.

## Authors’ contributions

Leyla Baykal Selçuk: Data curation; writing-original draft preparation; conceptualization; methodology.

Hande Ermiş Akkuş: Data curation; writing-original draft preparation.

Burak Akşan: Data curation; writing-original draft preparation.

Deniz Aksu Arıca: Data curation; writing-original draft preparation.

## Conflicts of interest

None declared.
