# Supplementary material for: Risankizumab and guselkumab for psoriasis: a 1-year real-world practice indirect comparison
Source: An Bras Dermatol. 2025 Jan 9;100(2):293–9. doi: 10.1016/j.abd.2024.05.005 (PMC11962891; doi:10.1016/j.abd.2024.05.005)
Supplement: Supplementary file 1 [file mmc1.docx]

**ABD-D-24-00204_ Material Supplementary**

**Material Supplementary**

|  | **Guselkumab** | | | | | | **Rısankızumab** | | | | | |
| --- | --- | --- | --- | --- | --- | --- | --- | --- | --- | --- | --- | --- |
|  | **PASI90** | | | **PASI100** | | | **PASI90** | | | **PASI100** | | |
|  | **Week 16** | **Week 24‒28** | **Week 52** | **Week 16** | **Week 24‒28** | **Week 52** | **Week 16** | **Week 24‒28** | **Week 52** | **Week 16** | **Week 24‒28** | **Week 52** |
| Ruggiero et al.[9] | 16 (69.6%) | ‒ | ‒ | 9 (39.1%) | ‒ | ‒ | 13 (61.9%) | ‒ | ‒ | 7 (33.3%) | ‒ | ‒ |
| Fougerosse et al.[11] | 50.6% | ‒ | ‒ | 38.3% | ‒ | ‒ | ‒ | ‒ | ‒ | ‒ | ‒ | ‒ |
| Benhadou et al.[12] | 62 (55.4%) | ‒ | ‒ | 36 (32.1%) | ‒ | ‒ | ‒ | ‒ | ‒ | ‒ | ‒ | ‒ |
| Gallozzo et al. [13] | ‒ | 72.5% | 78.9% | ‒ | 55.0% | 63.2% | ‒ | ‒ | ‒ | ‒ | ‒ | ‒ |
| Mastorino et al.[15] | ‒ | ‒ | ‒ | ‒ | ‒ | ‒ | 53% | ‒ | 82% | 32% | ‒ | 73% |
| Gkalapakotis et al.[16] | ‒ | ‒ | ‒ |  | ‒ | ‒ | 63.8% | ‒ | 82.4% | 44.7% | ‒ | 67.6% |
| Gargıulo et al.[17] | ‒ | ‒ | ‒ | ‒ | ‒ | ‒ | 55.7% | ‒ | 93.9% | 36.6% | ‒ | 61.1% |
| Hansel et al.[18] | ‒ | ‒ | ‒ | ‒ | ‒ | ‒ | ‒ | ‒ | 47 (85.5%) | ‒ | ‒ | 33 (60.0%) |
| Elgaard et al.[20] | 7 (46.7%) | ‒ | 7 (50.0%) | 1 (6.7%) |  | 7 (50.0%) | 7 (46.7%) |  | 4 (50.0%) | 6 (40.0%) | - | 4 (50.0%) |
| Gerdes et al.[21] | 48.2% | ‒ | 78.0% | 29.2% | ‒ | 50.4% | ‒ | ‒ | ‒ | ‒ | ‒ | ‒ |
| Galluzo et al.[25] | ‒ | ‒ | 68.7% | ‒ | ‒ | 51.1% | ‒ | ‒ | ‒ | ‒ | ‒ | ‒ |
| Baykal Selcuk et al. | 47 (95.9) | ‒ | 48 (98.0) | 17 (34.7) | ‒ | 27 (55.1) | 33 (80.5) | ‒ | 38 (92.7) | 14 (34.1) | ‒ | 22(53.7) |
